# Chirurgische Therapie der primär sklerosierenden Cholangitis: Erfahrungen aus 30 Jahren in einer monozentrischen Kohorte mit 173 konsekutiven Patienten

**DOI:** 10.1007/s00104-020-01197-5

**Published:** 2020-06-02

**Authors:** Vittorio Branchi, Tobias J. Weismüller, Taotao Zhou, Jonas Henn, Alexander Semaan, Tim R. Glowka, Maria Gonzalez-Carmona, Christian Strassburg, Jörg C. Kalff, Steffen Manekeller, Hanno Matthaei

**Affiliations:** 1 Klinik und Poliklinik für Allgemein‑, Viszeral‑, Thorax- und Gefäßchirurgie, Universitätsklinikum Bonn, Venusberg-Campus 1, 53127 Bonn, Deutschland; 2 Medizinische Klinik und Poliklinik I, Universitätsklinikum Bonn, Venusberg-Campus 1, 53217 Bonn, Deutschland

**Keywords:** Primär sklerosierende Cholangitis, PSC, Cholangiozelluläres Karzinom, Lebertransplantation, Leberresektion, HPB-Chirurgie, Primary sclerosing cholangitis, PSC, Cholangiocellular carcinoma, Liver transplantation, Liver resection, HPB-surgery

## Abstract

**Hintergrund:**

Wenngleich in Bezug auf Therapie und Krankheitsverständnis bei der primär sklerosierenden Cholangitis (PSC) in den vergangenen Jahren erhebliche Fortschritte zu beobachten sind, so ist bei Karzinom und „end-stage liver disease“ (ELD) eine Lebertransplantation (LTX) meist die einzige Chance auf Heilung. In seltenen Fällen ist eine Leberteilresektion (LR) zur kurativen Therapie des PSC-assoziierten Gallengangskarzinoms (CCC) möglich. Diese Operationen stellen für PSC-Patienten eine zusätzliche Belastung dar.

**Ziel:**

Auch aufgrund der Seltenheit der Erkrankung sind detaillierte Studien zur hepatopankreatikobiliären (HPB-)Chirurgie der PSC rar. Ziel dieser Arbeit ist es, die HPB-chirurgische Indikation und Prognose von PSC Patienten zu untersuchen.

**Patienten und Methoden:**

Es erfolgte eine monozentrische, retrospektive Kohortenstudie von 1990 bis 2020. In dieser wurden Patienten mit PSC eingeschlossen und in Bezug auf operationsassoziierte Faktoren sowie deren Prognose untersucht.

**Ergebnisse:**

Bei 62 Patienten (36 %) war eine PSC-bedingte, größere hepatopankreatikobiliäre Operation oder Exploration notwendig. Diese Patienten litten signifikant häufiger an einer chronisch entzündlichen Darmerkrankung (*p* < 0,019). Eine LTX erfolgte bei 46 dieser Patienten (73 %) aufgrund eines ELD. Eine LR erfolgte bei 8 Patienten (11 %). 9 Patienten wurden lediglich explorativ laparotomiert. Das Überleben in der LTX-Subgruppe war signifikant länger als nach LR bzw. explorativer Laparotomie (258 Monate; 95 %-Konfidenzintervall [CI] 210–306 vs. 88; 95 %-CI 16–161 vs. 13; 95 %-CI 3–23; *p* < 0,05).

**Diskussion:**

Ein großer Anteil der Patienten mit PSC muss aufgrund der Erkrankung operiert werden mit erheblichem Risiko für Morbidität und Mortalität. Bei fehlenden kurativen Therapieoptionen wären Frühdiagnosestrategien zu begrüßen, um das PSC-CCC im Frühstadium erkennen und behandeln zu können.

## Abstract (en)

**Background:**

In recent years substantial progress has been made in the treatment, surveillance and understanding of the pathogenesis of primary sclerosing cholangitis (PSC); however, in most cases liver transplantation (LTX) is still the only curative option for cancer or end-stage liver disease (ELD). In rare cases a partial liver resection is a possible curative treatment of a PSC-associated cholangiocellular carcinoma (CCC). These operations represent a significant additional burden for PSC patients.

**Objective:**

Due to the rarity of PSC detailed studies regarding hepato-pancreato-biliary (HPB) surgery are lacking. The aim of this study was to analyze the surgical indications and prognosis of PSC patients.

**Patients and methods:**

A single center retrospective cohort study from 1990 to 2020 was carried out. In this study patients with PSC were included and investigated with respect to factors associated with surgery and the prognosis.

**Results:**

As a consequence of PSC-associated conditions, in 62 patients (36%) a major HPB operation or explorative laparotomy was necessary. The prevalence of chronic inflammatory bowel disease was significantly higher in these patients (*P* < 0.019). An LTX was carried out in 46 patients (73%) because of ELD. A liver resection (LR) was performed in 8 patients (11%) and 9 patients only underwent an explorative laparotomy. The overall survival in the LTX subgroup was significantly longer than patients who underwent LR and explorative laparotomy (258 months; 95% confidence interval, CI 210–306 months vs. 88 months; 95% CI 16–161 months vs. 13 months; 95% CI 3–23 months; *p* < 0.05, respectively).

**Conclusion:**

The majority of patients with PSC have to be operated on because of the disease with substantial risks for morbidity and mortality. Curative treatment options are lacking, thus underlining the need for effective early detection and treatment strategies for PSC-CCC.

## Hintergrund

Die primär sklerosierende Cholangitis (PSC) ist eine chronisch fortschreitende Erkrankung der Leber und Gallenblase mit zunehmender Entzündung und Vernarbung der Gallengänge. Klinisch können sehr unterschiedliche Verläufe beobachtet werden [8, 29]. Die exakte Pathogenese und auch genetische Einflüsse hierbei sind noch nicht vollständig geklärt, wenngleich dieses Feld intensiv beforscht wird [1, 12, 20]. Die Hauptproblematik der Erkrankung entsteht durch Entzündung, Fibrose und damit Strukturverlust der intra- und extrahepatischen Gallengänge. Chronisch-rezidivierende Infektionen, Inflammation und Cholestase begünstigen dabei zum einen die Entartung des normalen Epithels über mittlerweile gut charakterisierte Vorläuferläsionen, den sog. biliären intraepithelialen Neoplasien (BilIN), bis hin zum PSC-assoziierten cholangiozellulären Karzinom (PSC-CCC; [14]). Das Risiko für dessen Entstehung ist bei der PSC bis zu 1000-fach erhöht im Vergleich zur Normalbevölkerung und die Tumoren zeigen oftmals ein besonders aggressives Verhalten [5, 26]. Zum anderen bedingen die rezidivierenden Cholangitiden eine progressive Zirrhose mit der Gefahr eines konsekutiven Leberversagens. Die häufig vorhandene Koexistenz einer chronisch entzündlichen Darmerkrankung (CED) ist eine zusätzliche Belastung für die Patienten, da hierbei das Risiko eines kolorektalen Karzinoms ebenfalls deutlich erhöht ist [29]. Vorsorgeuntersuchungen und der konservativen sowie endoskopischen Therapie kommt dabei ein besonderer Stellenwert zu. Da eine Heilung derzeit medikamentös nicht möglich ist, ergeben sich aber auch für die Viszeralchirurgie, neben der kolorektalen Chirurgie bei Karzinom, unterschiedliche gallenwegsassoziierte Operationsindikationen im Verlauf: Die historisch beschriebene operative Versorgung von Gallenwegsstenosen ist aufgrund exzellenter endoskopischer Therapie weitgehend obsolet [2]. Es kann aber z. B. bei den häufiger anzutreffenden Gallenblasenpolyen oder chronischer Cholezystitis eine Cholezystektomie indiziert sein [28]. Des Weiteren kann das CCC, sofern nicht initial inoperabel, eine Leberteilresektion (LR) und/oder Pankreatikoduodenektomie notwendig machen [7, 17]. Letztlich stellt die Lebertransplantation (LTX) die weiter einzig kurative Behandlungsoption bei der PSC dar mit z. T. exzellenten Langzeitergebnissen [9, 29].

Aufgrund der Seltenheit der Erkrankung sind größere und detaillierte Erhebungen zur Chirurgie der PSC in der Literatur rar. Zum besseren Verständnis des chirurgischen Therapiesektors im interdisziplinären Management der PSC erfolgte daher eine unizentrische Analyse unseres Patientenguts aus drei Jahrzehnten.

## Patienten und Methoden

### Patienten

Alle Patienten mit der Diagnose einer PSC (ICDK83.01), welche vom 01.01.1990 bis zum 31.12.2019 am Universitätsklinikum Bonn behandelt worden sind, wurden in die Studie eingeschlossen. Diese Studie erfolgt gemäß den ethischen Vorgaben der Universität Bonn und unter Berücksichtigung der Erklärung von Helsinki von 1975 (in der aktuellen, überarbeiteten Fassung). In allen Fällen erfolgte die Diagnose mittels Magnetresonanzcholangiopankreatikographie (MRCP) und/oder endoskopischer retrograder Cholangiopankreatikographie (ERCP). Patienten mit chronischer Gallenwegserkrankung oder Cholangitis, welche nicht eindeutig der PSC zugeordnet werden konnten, wurden ausgeschlossen.

Der überwiegende Anteil der Patienten war im Vorsorgeprogramm für PSC der Medizinischen Klinik I des Universitätsklinikums Bonn integriert. Des Weiteren wurden einige Patienten mit PSC zur Operationsevaluierung in unser tertiäres Referenzzentrum von extern überwiesen. Zur Abklärung einer chirurgischen Indikation erfolgte bei allen Patienten eine interdisziplinäre Konsultation bzw. bei Malignom eine Diskussion des Kasus in unserem wöchentlichen interdisziplinären Tumorboard. Die Studiengruppe der Operierten bildeten letztlich diejenigen Patienten, welche in der hepatopankreatobilären (HPB-)Chirurgie der Klinik und Poliklinik für Allgemein‑, Viszeral‑, Thorax- und Gefäßchirurgie für eine größere HPB-Operation evaluiert und operiert oder exploriert worden waren. Da der Fokus der Studie auf der gallengangsassoziierten Chirurgie lag, wurden kolorektalchirurgische Eingriffe, z. B. im Zusammenhang mit einer CED, nicht evaluiert. Demographische, klinische und behandlungsassoziierte Daten sowie Krankheitsverläufe wurden detailliert erhoben und ausgewertet. Zur Klärung der Frage, ob die Notwendigkeit einer größeren PSC-assoziierten abdominellen Operation (z. B. LTX bei Zirrhose und LR bei CCC) auf eine besondere klinische Subgruppe hinweist, wurde ein statistischer Vergleich dieser Patienten mit den nichtoperierten PSC-Patienten unternommen. Ein im Vorfeld geplantes Matching war hierbei letztlich nicht notwendig, da sich beide Gruppen hinsichtlich Alter und Geschlecht nicht signifikant voneinander unterschieden.

### Statistik

Die Analysen wurden mit der Software SPSS Statistics Version 22 (IBM, Armonk, New York, USA) durchgeführt. Es wurden die folgenden statistischen Tests angewandt: χ^2^-Test, Fisher-Exakt-Test, T‑Test. Die Überlebenskurven wurden nach der Kaplan-Meier-Methode erstellt und mittels Log-Rank-Test auf Signifikanz geprüft. Es wurde ein Konfidenzintervall (CI) von 95 % verwendet. Ein *p* < 0,05 wurde als statistisch signifikant gewertet.

## Ergebnisse

### Patientencharakteristika in der PSC-Kohorte des Universitätsklinikums Bonn

Von 173 Patienten der Bonner PSC-Kohorte war der überwiegende Teil männlich (*n* = 110; 64 %) und das Durchschnittsalter lag zum Zeitpunkt der Erstdiagnose bei 33 Jahren (Tab. 1). Während 9 Patienten (5 %) eine simultane Autoimmunhepatitis aufwiesen, betrug der Anteil der Patienten mit zusätzlicher CED 72 % (*n* = 125). Hiervon machte die Colitis ulcerosa (CU) den Großteil aus mit 84 % der CED (*n* = 105). Die Erstdiagnose der CED lag im Mittel 5 Jahre vor derjenigen der PSC. Die mittlere Zeit von PSC-Erstdiagnose bis zur HPB-Chirurgie betrug 9 Jahre. Ein PSC-CCC lag bei 17 Patienten (10 %) vor. Bis zur Entstehung waren im Durchschnitt 6 Jahre nach PSC-Erstdiagnose vergangen. Zu einem kolorektalen Karzinom (CRC) kam es bei ebenfalls 17 Patienten der Kohorte (10 %) und ein Doppelkarzinom (CCC und CRC) entstand bei 3 Patienten (2 %).

**Table 1:** 

		Alle Patienten *n* = 173	HPB-Chirurgie *n* = 62	Keine Chirurgie *n* = 111	*p*^a^
		(*n*, Median, oder Mittelwert [%, Range oder CI])	(*n*, Median, oder Mittelwert [%, Range oder CI])	(*n*, Median oder Mittelwert [%, Range oder CI])	
*Geschlecht*	M	110 (64)	41 (66)	69 (62)	0,603
	W	63 (36)	21 (34)	42 (38)	
*Alter PSC-Erstdiagnose (Jahre [Range])*		33 (9–73)	35 (15–65)	32 (9–73)	0,195
*AIH-Overlap*	Nein	164 (95)	61 (98)	103 (93)	0,112
	Ja	9 (5)	1 (2)	8 (7)	
*CU*	Nein	67 (39)	16 (26)	51 (46)	**0,014**
	Ja	105 (61)	45 (73)	60 (54)	
	Unbekannt	1 (1)	1 (2)	0 (0)	
*CED*	CU	105 (84)	45 (94)	60 (78)	**0,019**
	MC	13 (10)	3 (6)	10 (13)	
	COI	7 (6)	0 (0)	7 (9)	
*Alter CED-Erstdiagnose (Jahre [Range])*		28 (11–71)	28 (14–52)	28 (11–71)	0,656
*CED-Operation*	Nein	145 (84)	49 (79)	96 (86)	0,226
	Ja	26 (15)	12 (19)	14 (13)	
	Unbekannt	2 (1)	1 (2)	1 (1)	
*Cholangiokarzinom*	Nein	156 (90)	45 (73)	111 (100)	**<0,001**
	Ja	17 (10)	17 (27)	0 (0)	
*Alter CCC-Erstdiagnose*		48 (22–71)	48 (22–71)	–	–
*Zeit von PSC-Erstdiagnose zur CCC-Erstdiagnose (Jahre [Range])*		6 (0–18)	6 (0–18)	–	–
*Kolorektales Karzinom*	Nein	156 (90)	55 (89)	101 (91)	0,629
	Ja	17 (10)	7 (11)	10 (9)	
*Doppelkarzinom (CCC und CRC)*	Nein	170 (98)	59 (95)	111 (100)	**0,045**
	Ja	3 (2)	3 (5)	0 (0)	
*Follow-up Zeit (Monate [Range])*		63 (0–212)	57 (3–212)	38 (0–191)	**0,016**
*Mittleres Überleben nach PSC-Erstdiagnose (Jahre [CI])*		34 (30–39)	29 (23–35)	43 (39–47)	**0,004**
*Mittleres Überleben nach HPB-Chirurgie (Monate [CI])*		178 (124–232)	178 (124–232)	–	–

*AIH* Autoimmunhepatitis, *CED* chronische entzündliche Darmerkrankung, *CU* Colitis ulcerosa, *MC* Morbus Crohn, *COI* Colitis indeterminata, *CCC* Cholangiokarzinom, *CRC* kolorektales Karzinom, *CI* Konfidenzinterval,* PSC* primär sklerosierende Cholangitis

^a^χ^2^-Test, Fisher-Exakt-Test oder t‑Test entsprechend

### Vergleich operierte vs. nichtoperierte Patienten

Bei den Patienten in der operierten Kohorte lag im Vergleich zu den nichtoperierten Patienten häufiger eine CU vor (94 und 78 %, *p* = 0,014). Zudem lag bei den Patienten, welche operiert worden waren, signifikant häufiger ein CCC und (27 % vs. 0 %; *p* < 0,001) ein Doppelkarzinom CCC-CRC vor (5 % vs. 0 %; *p* = 0,045). Bei der durchgeführten Analyse ergaben sich keine statistisch signifikanten Unterschiede hinsichtlich Alter bei der PSC- oder CED-Erstdiagnose, Geschlecht und Autoimmunhepatitis(AIH)-Overlap.

### Chirurgische Therapie der PSC und assoziierte Überlebenszeiten

**Figure 1:**
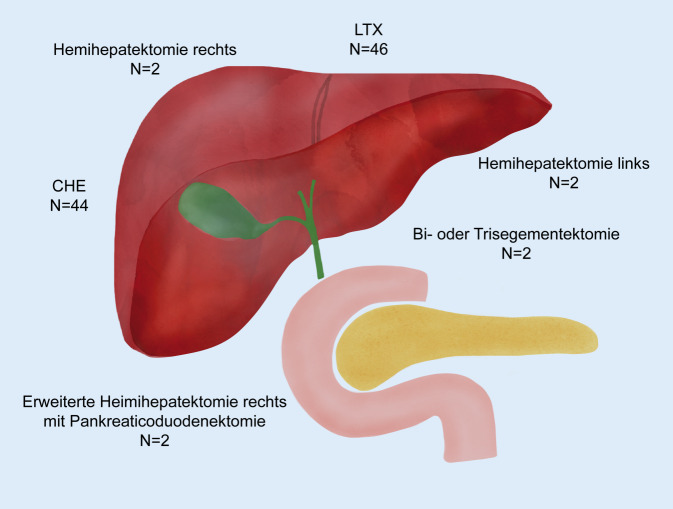

**Figure 2:**
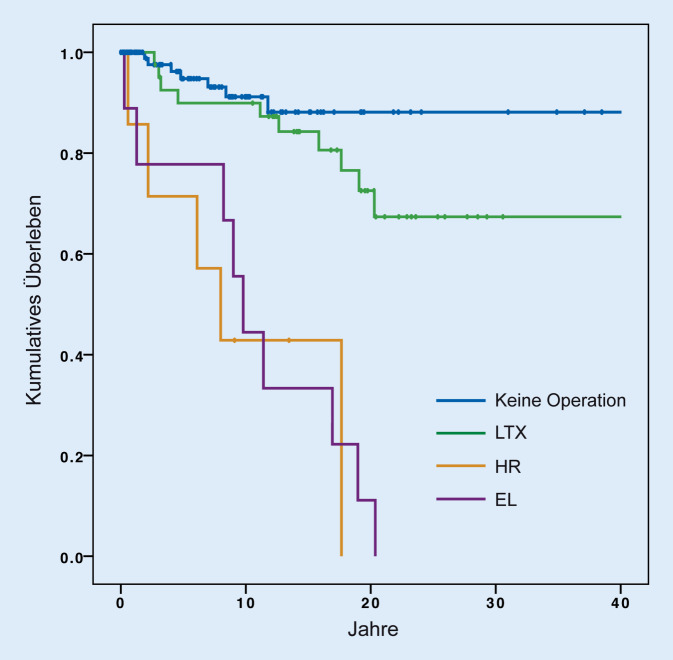

Bei 62 der 173 Patienten (35,8 %) wurde aufgrund der PSC eine größere HPB-Operation oder eine explorative Laparotomie aufgrund einer HPB-Indi\mathrm{kation durchgeführt (Tab. 2; Abb. 1). Die Lebertransplantation stellte dabei den häufigsten Eingriff dar (*n* = 46; 73 %). Diese wurde bei allen Patienten bei „end-stage liver disease“ (ELD) durchgeführt. Ein intrahepatisches CCC wurde bei 2 Patienten im Rahmen der Lebertransplantation festgestellt (7 %). Die LTX wurde in der überwiegenden Mehrheit mittels Vollorgan durchgeführt (*n* = 44; 96 %), während bei 2 Patienten (4 %) eine Split-LTX erfolgte. Es handelte sich in beiden Fällen um einen „extended right lobe“ (Lebersegmente 4 bis 8). Eine LR erfolgte bei 8 der operierten Patienten (11 %). Diese erfolgte bei 5 Patienten (62 %) aufgrund eines CCC und bei weiteren 3 Patienten (38 %) bei benigner Gallenwegsstenose. Hinsichtlich der resezierenden Verfahren wurde bei 2 Patienten (25 %) eine erweiterte Hemihepatektomie rechts mit simultaner Pankreatikoduodenektomie durchgeführt. Eine Hemihepatektomie rechts und eine Hemihepatektomie links wurden ebenfalls bei je 2 Patienten (25 %) durchgeführt. Eine Bisegmentektomie sowie eine Trisegmentektomie erfolgten jeweils bei einem Patienten (12,5 %). Lediglich explorativ laparotomiert wurden 9 Patienten (14 %). Dies erfolgte bei 8 Patienten (89 %) zum Staging bei CCC (und folglich Inoperabilität) und bei einem Patienten (11 %) zur Evaluation einer LTX. Bei einem Patienten wurde zunächst eine Hemihepatektomie rechts bei Verdacht auf CCC vorgenommen, welches sich histologisch nicht bestätigte. Sechs Jahre Später wurde der Patient bei ESLD transplantiert. Eine Cholezystektomie wurde bei 44 Patienten (25 %) als eigenständige Operation durchgeführt. Bei 30 Patienten wurde eine Gallenblasenentfernung simultan im Rahmen der LTX oder LR durchgeführt. Eine symptomatische Cholezystolithiasis war der Grund für die Cholezystektomie bei 25 Patienten (34 %). Präkanzeröse Läsionen wie die Porzellangallenblase oder Polypen stellten bei 5 Patienten (7 %) den Grund zur elektiven Cholezystektomie dar. Die akute Cholezystitis war bei 4 Patienten (5 %) die Indikation zur Gallenblasenentfernung. Das mittlere Gesamtüberleben nach PSC-Diagnose in der LTX-Kohorte betrug 37 Jahre (CI 31–43). Deutlich davon unterschied sich das Überleben der Patienten, die sich einer Leberresektion bzw. lediglich einer Exploration unterzogen (10 Jahre [CI 4–16] bzw. 11 Jahre [CI 6–15], *p* < 0,001; Abb. 2). Der häufigste Mortalitätsgrund nach LTX bei PSC war chronisches Leberversagen (*n* = 6, 60 %), gefolgt von Sepsis (*n* = 2, 20 %) und Multiorganversagen bei fortgeschrittener Malignität (*n* = 2, 20 %).

**Table 2:** 

		(*n* [%])	Follow-up (Monate [Range])	Mittl. Überleben nach PSC-Erstdiagnose (Jahre [CI])	Mittl. Überleben nach HPB-Chirurgie (Monate [CI])
*Major-HPB-Operationen/explorative Laparotomien*		*n* *=* *63*	–	–	–
*Transplantation*		46 (73)	96 (12–212)	37 (31–43)	258 (210–306)
Technik	Ganzes Organ	44 (96)	–	–	–
	Split-Leber	2 (4)			
*Leberresektion*		8 (11)	89 (13–169)	10 (4–16)	88 (16–161)
Indikation	Cholangiokarzinom	5 (62)	–	–	–
	Benigne Stenose	3 (38)			
Technik	Erw. HH rechts mit Whipple	2 (25)	–	–	–
	HH rechts	2 (25)			
	HH links	2 (25)			
	Trisegementektomie	1 (12,5)			
	Bisegementektomie	1 (12,5)			
*Explorative Laparotomie*		9 (14)	33 (3–109)	11 (6–15)	13 (3–23)
Indikation	Staging bei CCC	8 (89)	–	–	–
	LTX-Evaluation	1 (11)			
*Cholezystektomie*		*n* *=* *44*	–	–	–
Indikation	Cholezystolithiasis	25 (57)	–	–	–
	Porzellangallenblase/Polyp	5 (11)			
	Cholezystitis	4 (9)			
	Hydrops	1 (2)			
	Unbekannt	9 (20)			
Technik	Laparoskopische CHE	18 (41)	–	–	–
	Offene CHE	15 (34)			
	Unbekannt	11 (25)			

*HH* Hemihepatektomie, *CCC* Cholangiokarzinom, *LTX* Lebertransplantation, *CHE* Cholezystekomie, *CI* Konfidenzinterval, *PSC* primär sklerosierende Cholangitis

## Diskussion

Die primär sklerosierende Cholangitis ist eine seltene Erkrankung der Gallengänge mit schleichendem Beginn und sehr unterschiedlichen klinischen Verläufen [8, 29]. Zur medikamentösen Standardtherapie kommt nach wie vor Ursodeoxycholsäure (UDC) zum Einsatz [24]. Hierdurch wird der Gallefluss gefördert und die erhöhten Leberwerte, insbesondere die alkalische Phosphatase (AP) und die Gamma-Glutamyltransferase (GGT) gesenkt. Eine Effektivität hinsichtlich des Langzeitüberlebens konnte nicht prospektiv nachgewiesen werden. Möglicherweise profitieren laut Subgruppenanalysen aber Patienten mit gutem laborchemischem Ansprechen [16]. Auch hinsichtlich des cholestatisch bedingten Juckreizes kann durch UDC eine Besserung erzielt werden. Andere Medikamente, wie z. B. Colestyramin und Colestipol, haben diesbezüglich eine ähnliche, jedoch nicht so effiziente Wirkung im Vergleich zu UDC [11]. Einen großen Stellenwert nimmt die endoskopisch interventionelle Therapie dominanter Gallenwegsstrikturen ein, die bei konsequenter Durchführung das Langzeitüberleben der Patienten verbessert [22]. Im Verlauf kommt es dennoch bei den meisten Patienten zu rezidivierenden, z. T. schweren Entzündungsschüben in den Gallenwegen mit rezidivierenden Septitiden. Durch Entzündung und Cholestase entstehen dann die Wegbereiter für die chirurgischen Krankheitsbilder: In der Gallenblase kann es neben der akuten Cholezystitis zur Ausbildung von Gallensteinen und chronischer Cholezystitis kommen mit Entstehung von präkanzerösen Gallenblasenpolyen und Porzellangallenblase [28]. Ähnlich kann es im gesamten biliären Gangsystem zu dysplastischen Veränderungen kommen, den BilIN, bei denen eine Adenom-Karzinom-Sequenz beschrieben ist [14]. Diese Vorläuferläsionen des invasiven Gallengangskarzinoms können genau wie das CCC selbst zur Obstruktion an unterschiedlichen Stellen des abführenden Gallenwegsystems führen. Während bei allen tumorösen Prozessen eine operative Resektion evaluiert werden muss, so kann die fortschreitende biliäre Leberzirrhose eine Lebertransplantation notwendig machen (s. unten). Die Analyse der demographischen und krankheitsspezifischen Daten zeigte, dass unsere Patientengruppe aus drei Jahrzehnten diesbezüglich vergleichbar ist mit größeren Patientenkollektiven aus der Literatur [29]. Auch bei uns zeigten sich eine leichte männliche Dominanz und ein recht junges Alter bei PSC-Erstdiagnose. Auch die Entstehung eines CCC zeigte ein PSC-typisches Altersspektrum, welches deutlich unter dem Alter bei Entstehung eines sporadischen CCC lag. Auffälligkeiten der Gallenblase zählen zum charakteristischen Spektrum der PSC, wie Said et al. eindrucksvoll dokumentieren konnten. In einer konsekutiven Serie aus 286 Patienten mit PSC, welche an der Karolinska-Universität zwischen 1970 und 2005 untersucht wurden, fanden sich bei 41 % der Patienten eine oder mehrere Auffälligkeiten: Während Gallensteine und eine Cholezystitis jeweils bei einem Viertel der Patienten nachgewiesen werden konnten, so zeigte sich Letztere häufiger bei Patienten mit extrahepatischer PSC-Beteiligung als bei Patienten, welche lediglich die intrahepatischen Gallengänge affektiert hatten (30 % vs. 9 %). In dieser Studie wurde eine Raumforderung der Gallenblasenwand von im Mittel 21 mm bei 6 % der Patienten nachgewiesen, wohingegen 56 % der Patienten mit Gallenblasenkarzinom eine solche aufwiesen [23]. Präkanzerosen wie eine Porzellangallenblase oder Polypen waren mit 5 Patienten vereinzelt der Grund zur Operation. Wenngleich in der Literatur von einem erhöhten Risiko für die Entwicklung eines Gallenblasenkarzinoms bei PSC-Patienten berichtet wird, konnten wir diese Entität in unserer Kohorte nur bei einem Patienten beobachten. Bei diesem Patienten erfolgte die Cholezystektomie aufgrund sonographisch nachgewiesener Polypen. In der histopathologischen Aufarbeitung ergab sich das Vorliegen eines Adenokarzinoms der Gallenblase; daher folgte aus Radikalitätsgründen eine Bisegmentektomie. Es sei erwähnt, dass bildmorphologische Adenome der Gallenblase einer Beobachtung oder ggf. Resektion bedürfen. In einer Fallkontrollstudie von Sagwand et al. wurden Häufigkeit, Risikofaktoren und das Outcome von Gallenblasenpolypen bei Patienten mit PSC untersucht. Insgesamt 363 Patienten mit der etablierten Diagnose einer PSC und bildmorphologisch nachgewiesenen Polypen wurden mit PSC-Patienten ohne Gallenblasenpolypen verglichen. Die Häufigkeit der Gallenblasenpolypen betrug bei PSC-Patienten 10,6 % und war damit deutlich häufiger als in der normalen Bevölkerung, wo man diese nur selten antrifft. Von 16 Patienten mit Gallenblasenpolypen, welche sich einer Cholezystektomie unterzogen, hatten 4 Patienten eine „High-grade“-Dysplasie in ihrer Läsion und 6 Patienten ein invasives Adenokarzinom. Von den 6 Patienten mit invasivem Karzinom hatten 4 Patienten Läsionen, welche im maximalen Durchmesser größer als 10 mm waren. Zwei der Adenokarzinome waren allerdings mit 4 und 7 mm winzig, sodass die Autoren eine Cholezystektomie unabhängig von der Größe der Gallenblasenpolypen empfehlen [28]. Grundsätzlich gilt das laparoskopische Verfahren auch bei PSC-Patienten als Standard. Historisch gesehen zählte auch die chirurgische Entlastung des Gallenwegsystems bei Striktur mit z. B. biliodigestiver Drainage zu den Optionen, den Symptomen der fortschreitenden PSC zu begegnen. Mit dem Aufkommen und der steten Verbesserung der endoskopischen Therapie und im Hinblick auf die Morbidität in Assoziation mit der Chirurgie bei fortgeschrittener Lebererkrankung, ist diese Therapieoption in den letzten Jahren nur noch in wenigen Einzelfällen notwendig geworden. Die Lebertransplantation stellt die einzige definitive Therapie der PSC dar, wenngleich eine Rekurrenz der PSC beschrieben wird [9]. Auch wenn die klinischen Verläufe sehr unterschiedlich sind, so beträgt die Zeit von der Erstdiagnose bis zum Versterben bzw. zur Lebertransplantation wie in unserem Kollektiv oft nur wenige Jahre [27]. Obwohl für die Lebertransplantation ein Überlebensvorteil für Patienten bereits mit einem MELD-Score von >15 nachgewiesen werden konnte, muss bedingt durch den eklatanten Spenderorganmangel in Deutschland der MELD für Patienten für eine Leberallokation meist erheblich höher sein [19]. Für PSC-Patienten, bei denen das Fortschreiten der Erkrankung ja absehbar ist, ist die Lebertransplantation prognostisch gesehen auch schon vor einem weitgehenden Leberversagen sinnvoll, da die oftmals jungen Patienten eine geringe perioperative Morbidität zeigen. Unter bestimmten Umständen ist es PSC-Patienten möglich, zusätzliche MELD-Punkte angerechnet zu bekommen. Wiederholte Cholangitiden mit mehr als zwei Episoden einer Bakteriämie oder mehr als einer Episode einer Sepsis wären ein Grund für eine sog. „standard exception“ (SE; [6]). Schwerster therapierefraktärer Juckreiz kann ein Grund für eine „non-standard exception“ sein. Ein lokalisiertes CCC <3 cm im Durchmesser ohne Nachweis von intra- bzw. extrahepatischer Metastasierung qualifiziert im Rahmen von Studien ebenfalls für eine SE. Trotz dieser Optionen sind Wartezeiten oft lang. Als Alternative zum MELD-Score bietet sich in Einzelfällen eine Leberlebendspende an, welche sich als prognostisch besonders gut erwiesen hat [13]. In unserer Kohorte waren lediglich 2 Patienten mit einem cholangiozellulären Karzinom transplantiert worden. In beiden Fällen handelte es sich um einen Zufallsbefund nach histopathologischer Aufarbeitung der explantierten Lebern. Alle Patienten wurden bei progredientem Leberversagen transplantiert. Insgesamt ist die Auswahl der PSC-Patienten für eine Lebertransplantation aufgrund der schlechten Vorhersehbarkeit des Krankheitsverlaufs und der Dynamik der Karzinogenese extrem erschwert. Das MELD-System fördert im Zusammenhang mit der Organknappheit die Transplantation im meist weit fortgeschrittenen Krankheitsstadium. Es bleibt zu hoffen, dass zukünftig bessere prospektive Scores die Vorhersage von Krankheitsverläufen individuell erleichtern, sodass auf dieser Basis eine Lebertransplantation zeitlich besser geplant werden kann. Die fehlende Vorhersagbarkeit einer Karzinomentwicklung in der PSC stellt eine besondere Herausforderung dar: Das PSC-assoziierte Gallengangskarzinom zeigt im Vergleich zu den ohnehin sehr aggressiven sporadischen CCC noch einmal eine besonders schlechte Prognose. Da das Auftreten eines Gallenblasenkarzinoms wie in unserer Kohorte mit etwa 10–20 % deutlich erhöht ist im Vergleich zur Normalbevölkerung, ist ein Screening unabdingbar. Neben einer regelmäßigen Bildgebung mit Ultraschall und MRT, um raumfordernde Prozesse der Leber und Gallenwege zu identifizieren, wird eine regelmäßige ERCP mit Biopsieentnahme insbesondere bei Patienten mit morphologischen Auffälligkeiten der distaleren Gallengangsabschnitte empfohlen. Die Sensitivität und Spezifität dieser Untersuchung und auch diejenige der aktuellen Biomarkerstrategien sind relativ niedrig, sodass zuverlässige Frühdiagnosemöglichkeiten nicht existieren. Wenn ein invasives Karzinom erst einmal nachgewiesen ist, so ist dieses nur in wenigen Fällen operabel. Neben den 2 transplantierten Patienten wurden 5 Patienten aufgrund eines CCC reseziert. Bei anderen Patienten musste die Operation aufgrund eines fortgeschrittenen Tumorstadiums nach einer explorativen Laparotomie beendet werden mit letztlich fataler Konsequenz. Auch nach Resektion bleibt die Prognose infaust, wie das reduzierte Überleben in der resezierten Gruppe deutlich macht. Es stellte sich die Frage, ob die Notwendigkeit einer größeren PSC-assoziierten abdominellen Operation (z. B. LTX bei Zirrhose und LR bei CCC) auf eine besondere klinische Subgruppe hinweist. Im Weiteren verglichen wir daher die operierten und nichtoperierten Patienten. Die operierten litten häufiger an einer CED, insbesondere an CU, als mögliches Zeichen einer aggressiveren Entität und in der Literatur finden sich vermehrt Hinweise für diesen Zusammenhang. Es existiert die Hypothese, dass die CED in Assoziation mit PSC einen klinisch eigenständigen Erkrankungskomplex darstellt [10]. Durch die Heterogenität der überwiegend retrospektiven Analysen sind die Zusammenhänge hier noch nicht endgültig geklärt. Es scheinen in der klinischen Ausprägung der Erkrankungskomponenten zudem örtliche Unterschiede zu bestehen [3, 21]. Eines der Kerncharakteristika der Kombination PSC-CED ist allerdings, dass dieser Krankheitskomplex mit einem erhöhten Risiko für Malignität vergesellschaftet ist [15, 25]. In Übereinstimmung mit der Literatur konnte in unserem Gesamtkollektiv ein besonders hoher Anteil an CU-Patienten identifiziert werden, der in 94 % der operierten kulminierte [18]. Angenommen, in der operierten Kohorte handelt es sich um eine phänotypisch aggressivere Form der PSC, so lässt sich dies in unserem Kollektiv auch an einer höheren Rate an CCC sowie Doppelkarzinomen (CCC und CRC) belegen. Auch das CRC ist in ersterer Gruppe häufiger vertreten als in der Kohorte der Nichtoperierten, wenngleich sich hierfür keine statistische Signifikanz errechnen ließ. Die Assoziation zwischen Immunsuppression und Karzinomrisiko bei Patienten mit CED ist in der Literatur beschrieben, wenngleich noch viele Unklarheiten bezüglich der Ätiopathogenese bestehen. Das erhöhte Malignomrisiko in Bezug auf Immunsuppression bei diesen Patienten äußerst sich nach derzeitigem Kenntnisstand insbesondere auf nichtgastrointestinale Tumorerkrankungen wie Lymphom oder Leukämie sowie auf Hautkrebs und Urothelkarzinom [4]. Zusammengefasst scheint hier unter den Operierten eine besonders hohe Rate des vorbeschriebenen Phänotyps aus PSC und CED vorzuliegen, was noch einmal die deutlichere klinische Belastung in dieser Patientenpopulation begründet.

Einschränkend sei zu der obigen Studie zu erwähnen, dass es sich um retrospektiv erhobene Daten von Patienten handelt, welche über einen sehr langen Zeitraum monozentrisch behandelt wurden. Die Seltenheit der PSC bedingt dabei das Fehlen von Erfahrungen aus prospektiv randomisierten Studien. Durch die Unizentrizität lässt sich zudem ein Selektionsbias nicht vollständig vermeiden. Letztlich haben sich über die vergangenen drei Dekaden auch Gastroenterologie/Endoskopie, Viszeralchirurgie sowie auch das perioperative Management verbessert und sich auch das medizinische Leitlinienwerk entsprechend weiterentwickelt, sodass sich die Therapiestandards über den Untersuchungszeitraum gewandelt haben.

## Fazit für die Praxis

Wenngleich die PSC fast eine Dekade zuvor diagnostiziert wird, so ist im Verlauf bei einem Großteil der Patienten eine chirurgische Behandlung indiziert. Neben der Cholezystektomie als kleineren Eingriff steht dabei die Lebertransplantation zur definitiven Therapie im Fokus, da eine effektive medikamentöse Therapie weiterhin fehlt. Bei CCC stellt die Lebertransplantation eine Ausnahme dar. Hier ist meist eine Major-Hepatektomie gefordert, wobei bei einem Großteil bei fortgeschrittener Tumorerkrankung keine Resektion mehr erfolgen kann. Aus chirurgischer Sicht ist daher zur Vermeidung signifikanter Morbidität und Mortalität in Assoziation mit der PSC neben einer wirksamen Systemtherapie die Entwicklung effektiver Frühdiagnosestrategien ein essenzielles Ziel. Unsere Studie verdeutlicht, dass die komplexe Behandlung der PSC in einem erfahrenen interdisziplinären Zentrum erfolgen sollte.
